# Hic‐5 deficiency protects cerulein‐induced chronic pancreatitis via down‐regulation of the NF‐κB (p65)/IL‐6 signalling pathway

**DOI:** 10.1111/jcmm.14833

**Published:** 2019-12-03

**Authors:** Hao Chen, Peng Tan, Baolin Qian, Yichao Du, Ankang Wang, Hao Shi, Zhiwei Huang, Shiyao Huang, Tiancheng Liang, Wenguang Fu

**Affiliations:** ^1^ Department of Hepatobiliary Surgery The Affiliated Hospital of Southwest Medical University Luzhou China; ^2^ Academician (Expert) Workstation of Sichuan Province The Affiliated Hospital of Southwest Medical University Luzhou China; ^3^ Luzhou Municipal Hospital of Traditional Chinese Medicine Luzhou China; ^4^ Nuclear Medicine and Molecular Imaging Key Laboratory of Sichuan Province Luzhou China

**Keywords:** chronic pancreatitis, Hic‐5, NF‐κB, pancreatic stellate cells, triptolide

## Abstract

Chronic pancreatitis (CP), characterized by pancreatic fibrosis, is a recurrent, progressive and irreversible disease. Activation of the pancreatic stellate cells (PSCs) is considered a core event in pancreatic fibrosis. In this study, we investigated the role of hydrogen peroxide‐inducible clone‐5 (Hic‐5) in CP. Analysis of the human pancreatic tissue samples revealed that Hic‐5 was overexpressed in patients with CP and was extremely low in healthy pancreas. Hic‐5 was significant up‐regulated in the activated primary PSCs independently from transforming growth factor beta stimulation. CP induced by cerulein injection was ameliorated in Hic‐5 knockout (KO) mice, as shown by staining of tissue level. Simultaneously, the activation ability of the primary PSCs from Hic‐5 KO mice was significantly attenuated. We also found that the Hic‐5 up‐regulation by cerulein activated the NF‐κB (p65)/IL‐6 signalling pathway and regulated the downstream extracellular matrix (ECM) genes such as α‐SMA and Col1a1. Therefore, we determined whether suppressing NF‐κB/p65 alleviated CP by treating mice with the NF‐κB/p65 inhibitor triptolide in the cerulein‐induced CP model and found that pancreatic fibrosis was alleviated by NF‐κB/p65 inhibition. These findings provide evidence for Hic‐5 as a therapeutic target that plays a crucial role in regulating PSCs activation and pancreatic fibrosis.

## 1 INTRODUCTION

Chronic pancreatitis (CP) is a recurrent, progressive and irreversible inflammatory disease of the pancreas with a long course and poor prognosis. The main clinical manifestations of CP are abdominal pain, indigestion, steatorrhea and diabetes.^1^, ^2^ Importantly, patients with CP are at a higher risk for pancreatic cancer, one of the most devastating diseases.^3^, ^4^, ^5^ The main pathophysiological process underlying CP is the gradual replacement of the normal pancreatic tissue with atrophy and/or fibrosis, which leads to progressive loss of endocrine and exocrine regions, evident by pancreatic parenchymal atrophy and interstitial fibrosis.^6^, ^7^ Importantly, the mechanism underlying pancreatic fibrosis, a key pathological change in CP, is not fully understood.

The inflammatory microenvironment in CP includes a variety of cells, including pancreatic acinar cells, macrophages and pancreatic stellate cells (PSCs). In the normal pancreas, the few PSCs that are present in the connective tissue are quiescent. In this state, the PSCs are rich in vitamin A‐containing lipid droplets, and only a small amount of extracellular matrix (ECM) components are secreted.^8^ However, during CP, PSCs are activated as demonstrated by their morphology resembling fibroblasts. Additionally, the lipid droplets are either decreased in number or absent, there is active proliferation, and the secretion of ECM components such as type I and type III collagen is increased, which not only aggravates pancreatic fibrosis but also constitutes a barrier to drug penetration.^9^, ^10^ Therefore, activation of PSCs is considered a core event in pancreatic fibrosis.^11^, ^12^

Epithelial mesenchymal transition (EMT) is a biological phenomenon in which epithelial cells lose epithelial properties to obtain stromal cell phenotypes. Recent studies confirmed the role of EMT in fibrosis of organs such as the lungs and the liver.^13^, ^14^ Down‐regulation of epithelial markers such as E‐cadherin and cytokeratin and changes in cell morphology and migration are important features of EMT.^12^ Studies demonstrated that the processes underlying PSCs activation were similar to those observed during EMT,^15^ suggesting that the inhibition of PSCs activation is a promising target for an optimal treatment approach in CP.

Hydrogen peroxide‐inducible clone‐5 (Hic‐5), also known as transforming growth factor beta (TGF‐β)‐1‐induced transcript 1, is A LIM(Lin‐11, Isl‐1 and Mec‐3)‐containing adhesion scaffold protein with homology to paxillin.^16^ The N‐terminal region of Hic‐5 comprises four LD motifs (leucine‐aspartic acid motifs) that are rich in Leu and Asp, whereas the C‐terminal region comprises four LIM domains with two zinc fingers.^17^ Therefore, Hic‐5 not only has the ability to control cytoskeletal organization but also acts as an adaptor and nuclear receptor coactivator.^18^, ^19^ Hic‐5 was shown to be involved in the regulation of various cellular processes including proliferation, migration and differentiation, ageing, wound healing, vascular injury, tumorigenesis, steroid activity, apoptosis and signal integration.^19^

Recent studies revealed that Hic‐5 plays an important role in several fibrotic diseases. First, Hic‐5 was shown to be a key link in the formation of ECM in the abnormal glomeruli of glomerular sclerosis.^20^ Hic‐5 was also found to be positively correlated with the expression of the hepatic stellate cells activation marker α‐smooth muscle actin (α‐SMA) during liver fibrosis.^21^ Furthermore, Hic‐5 plays a key role in disorders such as intestinal fibrosis by regulating the differentiation of myofibroblasts and the expression of ECM proteins.^22^ However, the mechanism underlying Hic‐5‐mediated regulation of CP has not been determined.

In the current study, we investigated the effect of Hic‐5 by knocking out Hic‐5 in a mice model of CP and found that, in addition to changes in fibrosis‐related gene expression, the NF‐κB/p65 subunit and interleukin (IL)‐6 levels were decreased significantly as well. We also utilized primary PSCs isolated from mice to demonstrate the role of Hic‐5 in the regulation of PSCs activation, proliferation and migration. Finally, we investigated the effect of NF‐κB/p65 on CP development by inhibiting NF‐κB/p65 with triptolide, an immunosuppressant diterpenoid epoxide.

## 2 MATERIALS AND METHODS

### 2.1 Patients and tissue specimens

Tissue specimens were obtained from patients with CP, who underwent pancreatic resection, and from normal pancreatic tissue samples. Normal pancreas specimens were taken from two patients with pancreatic rupture caused by trauma, who had no underlying pancreatic disease. Both of them underwent pancreatic body and tail resection. Pathological results showed that the tail of the pancreas was normal pancreatic tissue. In all cases, freshly removed tissue samples were fixed in 4% paraformaldehyde solution for 24 hours and were paraffin‐embedded for histological analysis. Besides, a portion of the tissue samples was snap‐frozen in liquid nitrogen immediately upon surgical removal and maintained at −80°C until use. Our researchers complied with the International Ethical Guidelines for Biomedical Research Involving Human Subjects (CIOMS). After being informed of the aims and procedures in this study, which was approved by the Ethics Committee of the Affiliated Hospital of Southwest Medical University (Luzhou, China), all participants signed informed consents.

### 2.2 Construction of experimental model

Hic‐5 KO mice are produced by Beijing Biocytogen Co., Ltd. Wild‐type (WT) and systemic Hic‐5 knockout (Hic‐5 KO) mice (C57BL/6 background) were maintained under specific pathogen‐free conditions in the laboratory animal centre of Southwest Medical University. Experiments were performed with age‐and‐sex matched mice at 8‐12 weeks of age. Chronic pancreatitis (CP) was induced by repeated intraperitoneal injections of cerulein (Med Chem Express, MCE), 50 μg/kg hourly, by a modification of the method described by Westphalen et al^23^ Six hourly injections given in one day constituted one treatment. Treatments were given every other day for a total of six weeks. Triptolide‐treated model was induced in mice through intraperitoneal injection of triptolide (100 µg/kg, Med Chem Express, MCE) after two‐week cerulein treated, by a modification of the method described by Li et al^24^ Injections were given six times per week for a total of four weeks. Sex‐and‐age matched control mice received comparable injections of saline. Each pancreas was removed, weighed and divided into three sections. All experiments were approved by the regional Animal Study Committees and performed according to the institutional guidelines stipulated by The Affiliated Hospital of Southwest Medical University.

### 2.3 Cells isolation

Pancreatic stellate cells were isolated from mice pancreas by collagenase digestion and Nycodenz density gradient centrifugation. The pancreas of three mice were pooled to get enough cells for the experimental setting. PSCs were isolated from pancreas of wild‐type and Hic‐5 KO C57BL/6 mice at 8‐12 weeks of age by the method described by Apte et al^25^ Isolated PSCs were washed with GBSS and resuspended in DMEM (Invitrogen) containing 10% characterized FBS (GIBCO) and antibiotics (penicillin 100 U/mL and streptomycin 100 mg/mL, Invitrogen). Cells were maintained in a humidified atmosphere of 5% CO_2_ at 37°C. After reaching 80% confluence, cells were briefly trypsinized (0.25% Trypsin‐EDTA, Invitrogen) and subcultured. Cells were used before the first passage. Primary pancreatic acini were isolated from mice pancreas by collagenase digestion under sterile conditions, as previously reported.^26^ Isolated acini were cultured in RPMI‐1640 medium containing penicillin (50 U/mL) and streptomycin (50 μg/mL) supplemented with 10% serum at 37°C in a 5% CO_2_ atmosphere.

### 2.4 RNA isolation, reverse‐transcription and quantitative real‐time polymerase chain reaction (qRT‐PCR)

RNA isolation and the PCR amplification conditions were followed as manufacturer's instructions (Toyobo, Osaka, Japan). Real‐time PCR was performed to quantify the expression of several genes in cultured cells and pancreatic tissues using a real‐time PCR detection system (LightCycler 96, Roche). Samples were run in triplicates, expression values were normalized against RPL‐13A genes,^27^, ^28^ and fold changes were calculated to the control group using the 2^−ΔΔCT^ method. Gene‐specific primers were described in Table 1.

**Table 1:** Sequence (5′–3′) of primers used in this study

Gene name	Forward primer	Reverse primer
*Hic‐5*	ATGTCACGGTTAGGGGCTC	GGCTTGCATACTGTGCTGTATAG
*TGF‐β*	CTCCCGTGGCTTCTAGTGC	GCCTTAGTTTGGACAGGATCTG
*Col1a1*	ACGCATGGCCAAGAAGAC	GGTTTCCACGTCTCACCATT
*α‐SMA*	TGACAGGATGCAGAAGGAGA	CCACCGATCCAGACAGAGTA
*Col3a1*	AACCTGGTTTCTTCTCACCCTTC	ACTCATAGGACTGACCAAGGTGG
*TIMP1*	GCAAAGAGCTTTCTCAAAGACC	CTCCAGTTTGCAAGGGATAGAT
*NF‐κB/p65*	ACTGCCGGGATGGCTACTAT	TCTGGATTCGCTGGCTAATGG
*IL‐6*	TTCTCTGGGAAATCGTGGAAA	TGCAAGTGCATCATCGTTGT
*RPL‐13A*	GAGGTCGGGTGGAAGTACCA	TGCATCTTGGCCTTTTCCTT

### 2.5 Western blot analysis

Tissues and cells were lysed in RIPA lysis buffer (Catalog No. P0013B, Beyotime). The lysis was centrifuged, and the supernatant was collected for Western blot analysis. Samples of protein were resolved on SDS‐PAGE and transferred onto PVDF membranes (Catalog No. ISEQ00010, Millipore). The membranes were incubated with the following primary antibodies overnight at 4°C: anti‐Hic‐5 (Catalog No. ab42476, 1:1000, abcam), anti‐α‐SMA (Catalog No. ab5694, 1:500, abcam), anti‐Col1a1 (Catalog No. GB11022, 1:1000, Servicebio), anti‐IL‐6 (Catalog No. GB11117, 1:1000, Servicebio), anti‐NF‐κB/p65 (Catalog No. 10745‐1‐AP, 1:1000, Proteintech), anti‐Vimentin (Catalog No. GB11192, 1:1000, Servicebio) and anti‐GAPDH (Catalog No. 60004‐1‐Ig, 1:5000, Proteintech). Then, the membranes were incubated with horseradish peroxidase‐conjugated secondary antibodies for 1 hour at room temperature. The following secondary antibodies conjugated to horseradish peroxidase: Affinipure goat antimouse IgG (Catalog No. SA00001‐1, 1:5000, Proteintech) and Affinipure goat anti‐rabbit IgG (Catalog No. SA00001‐2, 1:5000, Proteintech).

### 2.6 Cell proliferation assay

PSCs were seeded at a density of 5000 cells per well in E‐Plate L8 (Catalog No. 00300600850, ACEA), activated with 5 ng/mL TGF‐β. Cell proliferation was analysed with iCELLigence RTCA Analyzer (ACEA, Hangzhou, China). Cell index signals were obtained automatically using the RTCA Analyzer.

### 2.7 Wound healing assay

Cells were seeded into a 6‐well plate and allowed to grow to full confluence. To study migration, a scratch was made on the culture plate using a 200 μL pipette tip fixed in a custom‐made holder. The cells were washed to remove all detached cells and incubated with fresh serum‐free medium. Cells were observed and pictures were taken at indicated times. Images were analysed by ImageJ software to calculate the area of the scratch and represented as the percentage of wound closure.

### 2.8 Transwell migration assay

Migration assays were carried out by using Transwell chambers with pore polycarbonate filters (8.0 mm; Corning), which were inserted in a 24‐well plate. In brief, cells in serum‐free medium were added to the upper chamber. Medium with 10% foetal bovine serum as the chemoattractant was placed in the lower chamber. The plates were incubated for 24 hours at 37°C in 5% CO_2_; afterwards, cells that did not migrate via the pores were removed by wiping them with a cotton swab. Cells left on the lower surface of the membrane were stained with 0.1% crystal violet. At last, we cleaned the upper chamber and inverted the chamber, counted the number of cells on the lower surface of the membrane.

### 2.9 Haematoxylin and eosin staining, Masson's trichrome staining and Sirius Red staining

Pancreatic tissues from killed mice were immediately fixed in 4% paraformaldehyde for paraffin embedding. Paraffin sections were used for haematoxylin and eosin (H&E), Masson's trichrome staining and Sirius Red staining. H&E staining was performed using a commercial kit (Catalog No. C0105, Beyotime) under the manufacturer's instructions. Masson's trichrome staining was performed following the manufacture's protocol with a staining kit (Catalog No. G1340, Solarbio). Sirius Red staining of paraffin‐embedded pancreas sections was performed with a staining kit (Catalog No. S8060, Solarbio). Fibrotic areas, which appeared blue under Masson's trichrome staining and Red under Sirius Red staining, were quantified by ImageJ 1.43 (W. S. Rasband, ImageJ, U. S. National Institutes of Health). The non‐pancreatic regions were subtracted when calculating the total area of pancreatic tissue in the selected field. The percentage of fibrotic area was calculated from the ratio of fibrotic tissue to total pancreatic tissue.

### 2.10 Immunohistochemistry

Antigen retrieval was performed by heating slides in the microwave for 20 minutes in 0.01 mol/L citrate buffer (pH 6.0), and 3% hydrogen peroxide was added for 10 minutes to quench peroxidase activity. Sections were treated with normal goat serum, followed by incubation overnight with anti‐Hic5 antibody (Catalog No. ab42476, 1:100, abcam) or anti‐α‐SMA antibody (Catalog No. ab5694, 1:100, abcam) at 4°C. Sections were then rinsed with phosphate‐buffered saline (PBS), incubated with secondary antibody for 1 hours, stained with diaminobenzidine and counterstained with haematoxylin. Sections were dehydrated and sealed, then examined using a microscope (Olympus).

### 2.11 Immunofluorescence

The cells were permeabilized in 0.1% Triton‐X/PBS for 20 minutes at room temperature. After incubation with anti‐Hic5, anti‐NF‐κB/p65 or anti‐α‐SMA for overnight at 4°C in a dark place, the cells were washed with PBS for three times. Then, the cells were incubated with secondary antibodies for 1 hour, and 4′6‐diamino‐2‐phenylindole (DAPI, Beyotime) was added to stain the cell nuclei. The cells were detected by a laser scanning confocal microscope (Olympus).

### 2.12 ELISA

Serum amylase and IL‐6 levels were assayed by ELISA kit according to the manufacturer's instructions (Catalog No. MU20882, MU30044, R&D Systems).

### 2.13 Statistical analysis

Animals were randomly allocated to control and treatment groups. All data were collected from three independent experiments. All data were expressed as means ± the standard error of the mean. Statistical analysis was performed with GraphPad Prism 6.0 software, and analysis of Student's *t* test was used to evaluate statistical significance. Differences are considered significant when *P < .05, **P < .01, ***P < .001 and *ns P* > .05.

## 3 RESULTS

### 3.1 Pancreatic Hic‐5 expression is induced in CP

**Figure 1:**
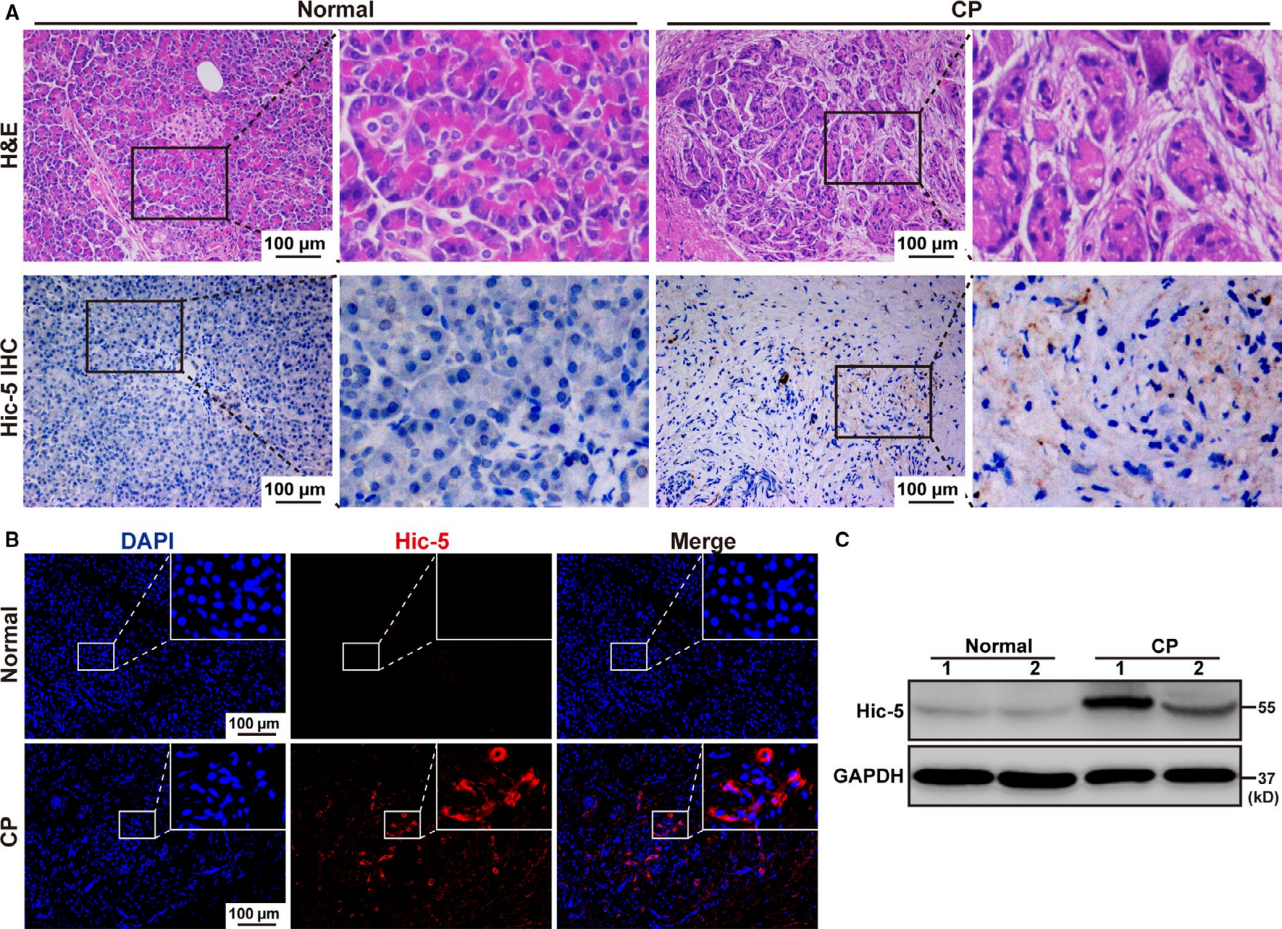
Hic‐5 expression is enhanced in the pancreas of patients with CP. A, Representative images of H&E staining and immunohistochemistry for Hic‐5 in the pancreas of normal and CP patients. Scale bar, 100 μm. High‐magnification images are shown next to the graph. B, Representative images of the immunofluorescence for DAPI and Hic‐5 in the pancreas of normal and CP patients. Scale bar, 100 μm. The merged images are shown next to the graph. C, Western blotting for Hic‐5 in the pancreas of normal and CP patients. *P < .05, **P < .01, ***P < .001, by two‐tailed, unpaired Student's *t* test. (n = 2)

**Figure 2:**
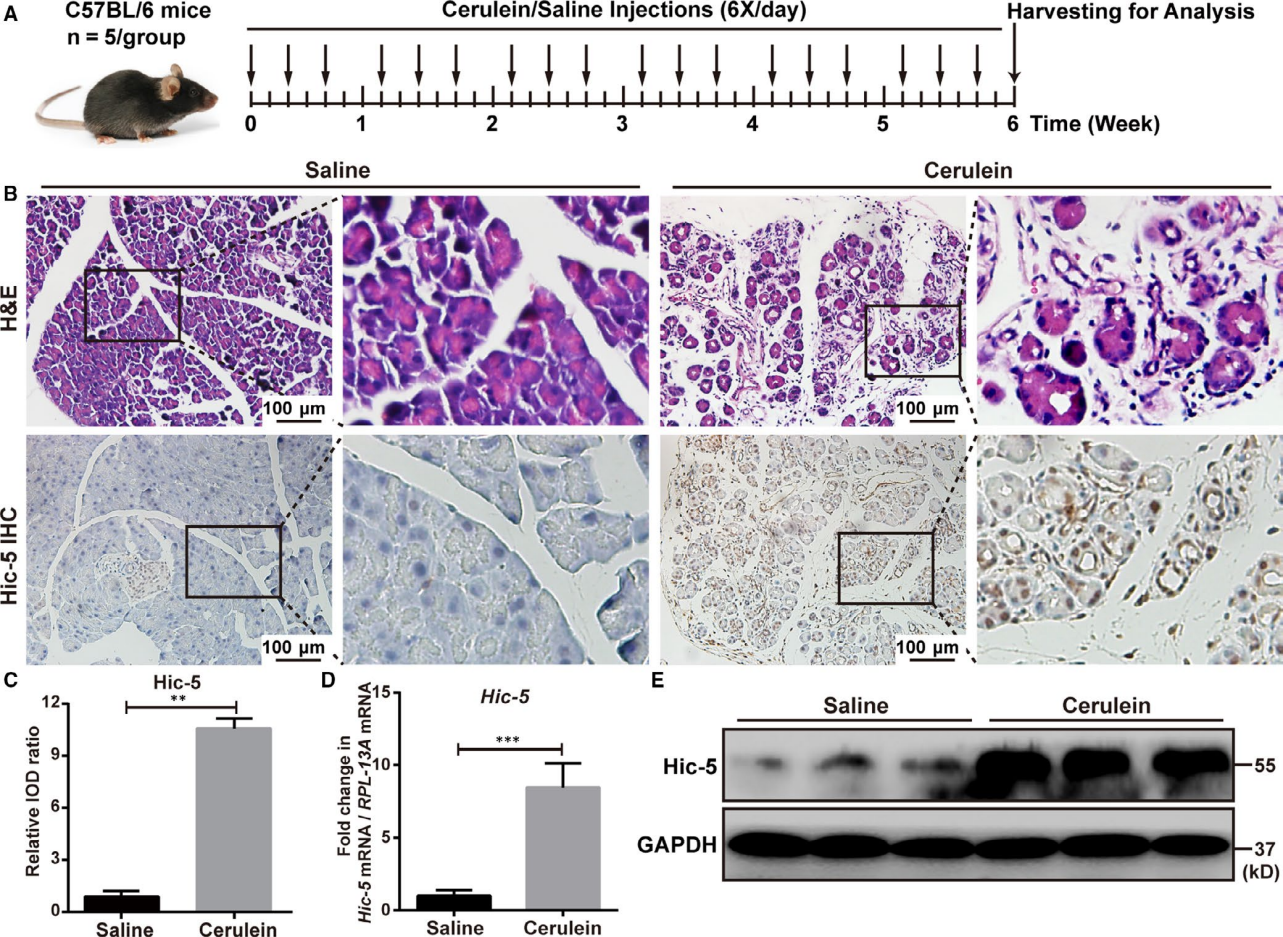
Hic‐5 expression is enhanced in the pancreas in a mice model of CP. A, The schedule of mice experimental procedure is shown by a diagram. CP was induced via intraperitoneal injection of cerulein (50 μg/kg). Control group was treated with saline alone. B, Representative images of H&E staining and immunohistochemistry for Hic‐5 in the pancreas of normal and CP mice. Scale bar, 100 μm. High‐magnification images are shown next to the graph. C, Quantification of the immunohistochemistry for Hic‐5 in the pancreas of normal and CP mice. D, Quantitative real‐time PCR for Hic‐5 in the pancreas of normal and CP mice. E, Western blotting for Hic‐5 in the pancreas of normal and CP mice. *P < .05, **P < .01, ***P < .001, by two‐tailed, unpaired Student's *t* test. (n = 5)

Since most patients with chronic pancreatitis who visit our hospital do not need surgery, we regret that there are only two specimens of chronic pancreatitis. We used these specimens for haematoxylin and eosin (H&E) staining and found that compared to the normal person, the necrotic and inflammatory areas in pancreas of patients with CP were markedly increased (Figure 1A). Next, we evaluated Hic‐5 expression in the pancreas of normal person and CP patients and found that the protein expression level of Hic‐5 in the pancreas was significantly increased in the patients with CP compared to the normal person by immunohistochemistry and immunofluorescence (Figure 1A,1). Furthermore, Western blot analysis confirmed that the protein expression levels of Hic‐5 in the pancreas were markedly increased in the CP patients compared to the normal person (Figure 1C). We induced CP in C57BL/6 mice using cerulein, as previously described (Figure 2A), and confirmed the induction of CP by haematoxylin and eosin (H&E) staining (Figure 2B). Next, we evaluated Hic‐5 expression in the pancreas of untreated and CP mice and found that the protein expression level of Hic‐5 in the pancreas was significantly increased in the mice with CP compared to the untreated mice by immunohistochemistry (Figure 2B,2). Furthermore, qRT‐PCR and Western blot analysis confirmed that the mRNA and protein expression levels of Hic‐5 in the pancreas were markedly increased in the CP mice compared to the untreated mice (Figure 2D,2).

### 3.2 Knockout of Hic‐5 attenuates cerulein‐induced CP in vivo

**Figure 3:**
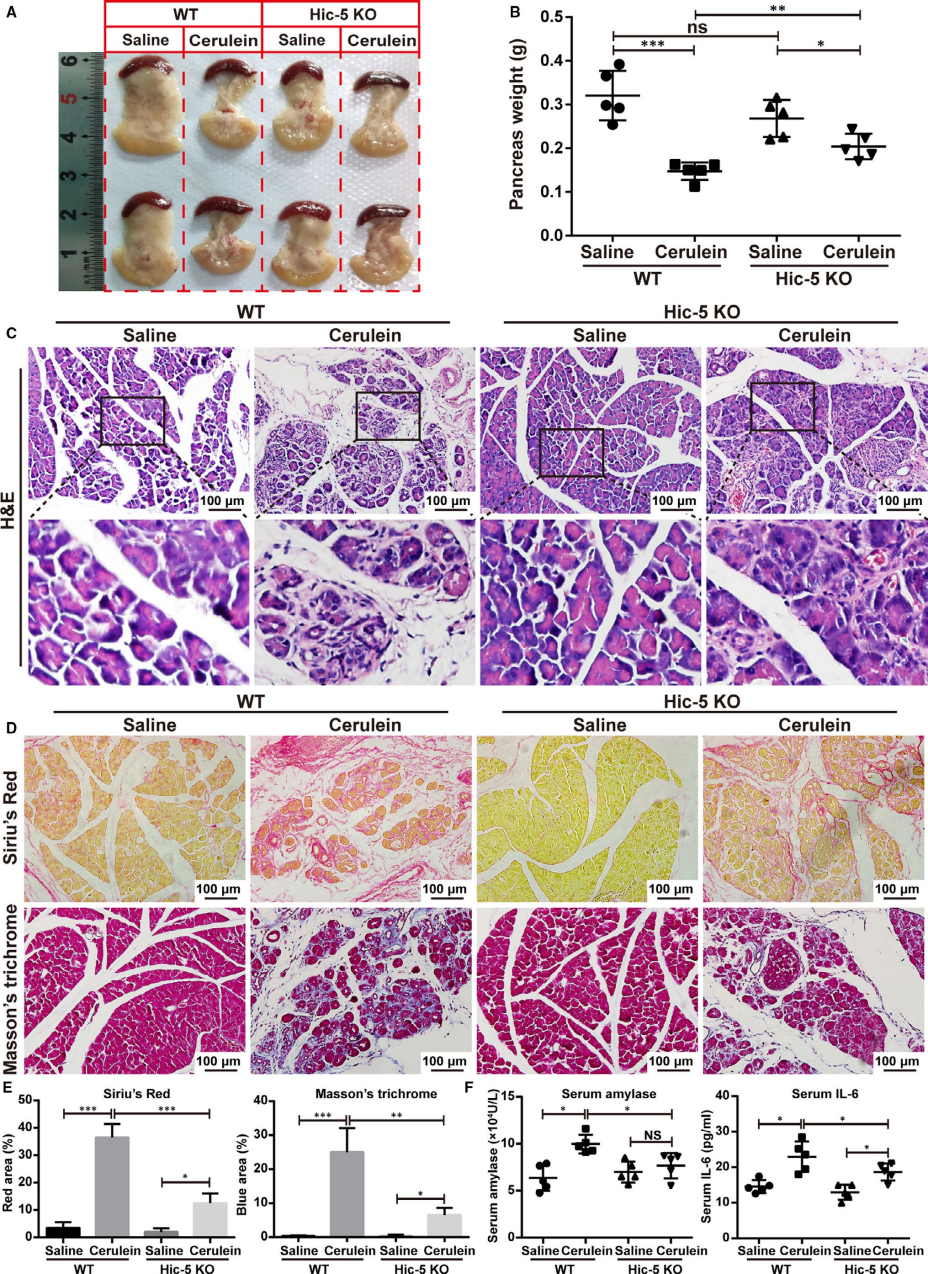
Knockout of *Hic‐5* attenuates cerulein‐induced CP in vivo. A, Typical gross appearance of the pancreas. B, Quantification of the pancreas weight of wild‐type and Hic‐5 knockout mice with or without cerulein induction. C, Representative images of H&E‐stained pancreas of the normal and CP mice. Scale bar, 100 μm. High‐magnification images are shown below. D, E, Representative images (D) and quantification (E) of Sirius Red and Masson's trichrome staining which represent fibrosis. F, Serum amylase and IL‐6 levels were assayed by ELISA. *P < .05, **P < .01, ***P < .001, by two‐tailed, unpaired Student's *t* test. (n = 5)

To investigate the contribution of Hic‐5 to the development of CP, we induced CP in wild‐type and Hic‐5 KO mice by cerulein. After treatment of mice with cerulein, we found that the pancreas size and weight in the Hic‐5 KO mice were significantly increased compared to the wild‐type mice (Figure 3A and 3). Histological examination of the H&E‐stained pancreatic sections indicated that the necrotic and inflammatory areas were significantly reduced in the Hic‐5 KO mice compared with the wild‐type mice after cerulein treatment (Figure 3C). Sirius Red and Masson's trichrome staining used for morphometric analysis of the pancreatic fibrosis revealed that the pancreatic fibrosis was significantly reduced in the Hic‐5 KO mice compared with the wild‐type mice after treatment with cerulein, based on both staining methods (Figure 3D,3). We next analysed serum levels of amylase and IL‐6 by ELISA and confirmed that the levels of amylase and IL‐6 in the peripheral blood of the Hic‐5 KO mice treated with cerulein were significantly lower than those of the wild‐type mice treated with cerulein (Figure 3F).

### 3.3 Knockout of Hic‐5 decreases the expression of cerulein‐induced pancreatic fibrosis‐related factors and NF‐κB/p65 in vivo

**Figure 4:**
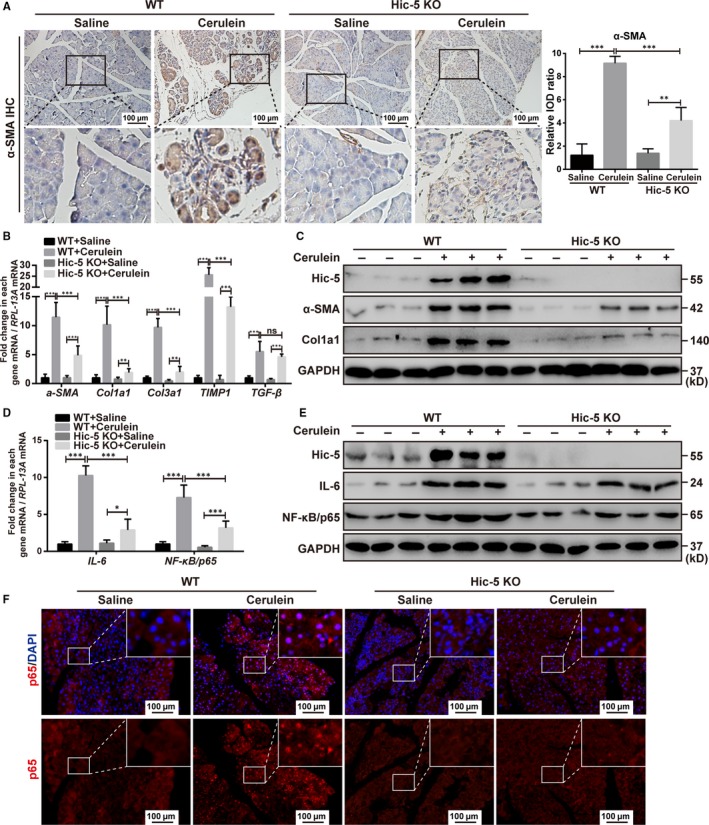
Knockout of Hic‐5 decreases the expression of cerulein‐induced pancreatic fibrosis‐related factors and NF‐κB/p65 in vivo. A, Representative images and quantification of the immunohistochemistry for α‐SMA in WT mice and Hic‐5 KO mice with CP induced by cerulein. Scale bar, 100 μm. High‐magnification images are shown below. B, D, Quantitative real‐time PCR of the pancreatic tissue for α‐SMA, *Col1a1*, *Col3a1*, *Timp1* and TGF‐β (B), and IL‐6 and NF‐κB/p65 (D). C, E, Representative Western blot analyses for Hic‐5, α‐SMA, and Col1a1 (C), and IL‐6 and NF‐κB/p65 (E) in the pancreas of WT and Hic‐5 KO mice. F, Representative images of the immunofluorescence for NF‐κB/p65 in WT mice and Hic‐5 KO mice with CP induced by cerulein. Scale bar, 100 μm.*P < .05, **P < .01, ***P < .001, by two‐tailed, unpaired Student's *t* test. (n = 5)

Immunohistochemical analysis of the pancreas sections indicated that the expression of α‐SMA, a marker for activated PSCs,^29^ was significantly decreased in the pancreas of Hic‐5 KO mice treated with cerulein compared with the wild‐type mice treated with cerulein (Figure 4A). We evaluated the mRNA levels of fibrosis‐associated genes, including *α‐SMA, Col1a1, Col3a1*, *Timp1* and *TGF‐β*, in the pancreas by qRT‐PCR. The expression levels of *α‐SMA, Col1a1, Col3a1* and *Timp1* in the pancreas of the Hic‐5 KO mice were significantly lower than those of the wild‐type mice, following cerulein treatment (Figure 4B). Interestingly, there was no difference in the mRNA expression of TGF‐β between the wild‐type and the Hic‐5 KO mice after cerulein treatment (Figure 4B). In addition, Western blot analysis confirmed that the expression of α‐SMA and Col1a1 in the pancreas of the Hic‐5 KO mice was significantly lower than that of the wild‐type mice after cerulein treatment (Figure 4C). These results indicated that Hic‐5 deficiency contributed to the improvement observed in mice with cerulein‐induced CP. Besides, we evaluated the mRNA and protein levels of the NF‐κB/p65 subunit and IL‐6 in the pancreas by qRT‐PCR and Western blot analysis. We found that the expression levels of NF‐κB/p65 and IL‐6 were higher in the pancreas of the cerulein‐treated wild‐type mice compared with the untreated mice and that their expression levels were significantly decreased in the pancreas of the cerulein‐treated Hic‐5 KO mice (Figure 4D,4). Also, as shown in the result of immunofluorescence (Figure 4F) and immunohistochemistry (Figure S1A) for NF‐κB/p65, the total expression level and nuclear expression level of NF‐κB/p65 in the pancreas of cerulein‐treated Hic‐5 KO mice were significantly lower than that of cerulein‐treated WT mice (Figures 4F and S1A). Our immunohistochemistry for IκB‐α suggested there were no significant differences in IκB‐α expression between cerulein‐treated Hic‐5 KO mice and cerulein‐treated WT mice (Figure S1B).

### 3.4 Hic‐5 deficiency inhibits TGF‐β‐induced PSCs activation and NF‐κB/p65 expression

**Figure 5:**
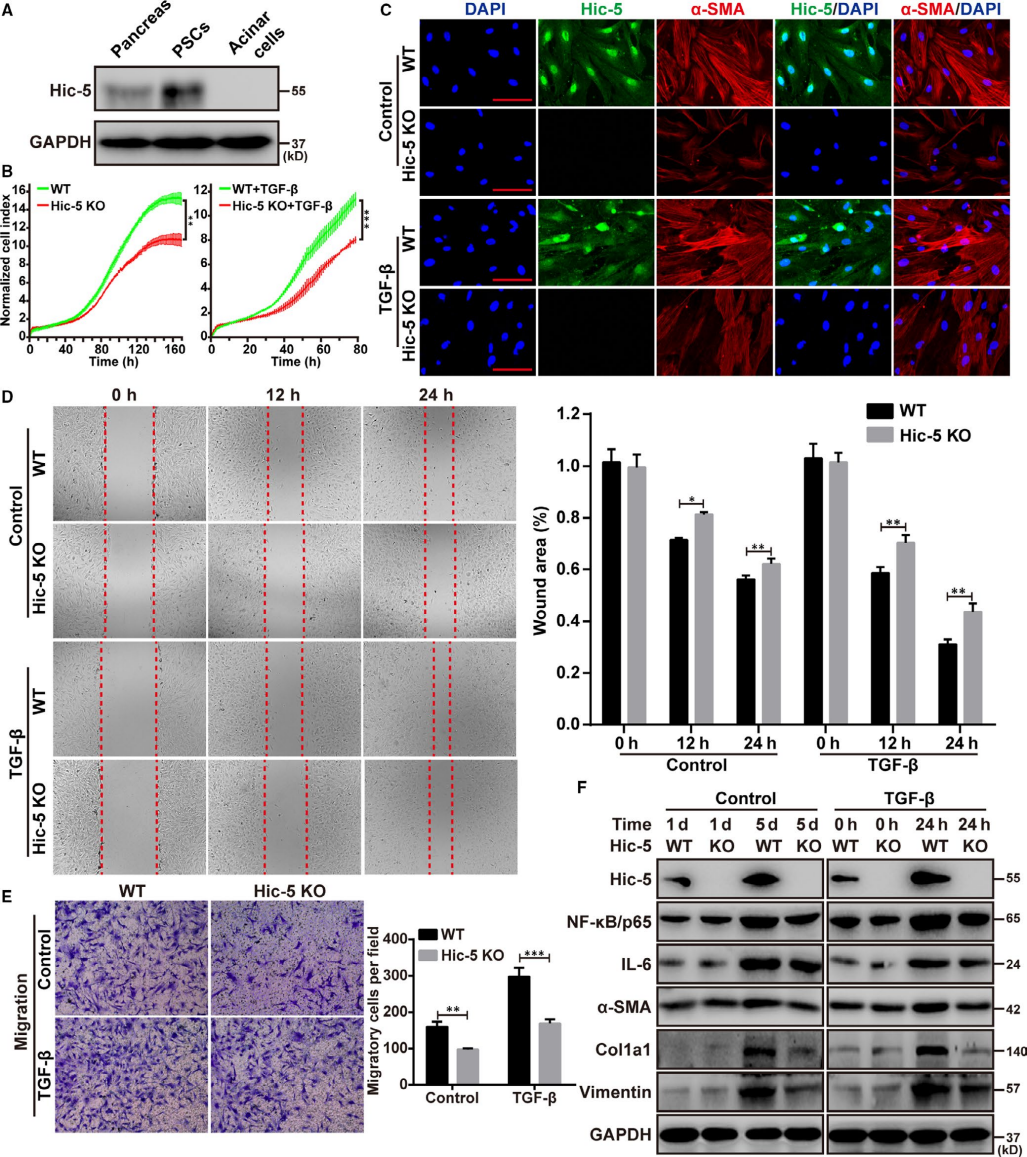
Hic‐5 deficiency inhibits TGF‐β‐induced PSCs activation and NF‐κB/p65 expression. A, Representative Western blots for Hic‐5 in pancreas, acinar cells and pancreatic stellate cells. B, Cell proliferation curve analysed by iCELLigence RTCA analyzer. C, Immunofluorescence staining for Hic‐5 (green) and α‐SMA (red) in primary PSCs isolated from WT or Hic‐5 KO mice with or without TGF‐β stimulation. Scale bar, 100 μm. D, E, Representative microscopic images and quantification showing the migration ability of PSCs isolated from WT or Hic‐5 KO mice with or without TGF‐β stimulation. F, Representative Western blots for Hic‐5, NF‐κB/p65, IL‐6, α‐SMA, Col1a1 and vimentin in PSCs from WT and Hic‐5 KO mice with or without TGF‐β stimulation. *P < .05, **P < .01, ***P < .001, by two‐tailed, unpaired Student's *t* test. (n = 5)

Previous studies have reported that Hic‐5 was expressed only in hepatic stellate cells but not in hepatocytes cells, hepatic macrophages (kupffer cells) and liver sinusoid endothelial cells.^21^ To investigate which cell types in the pancreas are responsible for Hic‐5 expression, we isolated mouse primary pancreatic stellate cells and acinar cells. Then, we used Western blot analysis to examine Hic‐5 expression in pancreas, acinar cells and pancreatic stellate cells. After culturing for 12 hours, Hic‐5 expression was detected in pancreatic stellate cells, but not in acinar cells (Figure 5A). The activation of PSCs plays a crucial role in promoting ECM production and pancreatic fibrosis.^29^ Therefore, we assessed whether Hic‐5 was required for the activation of PSCs in vitro in primary PSCs isolated from the pancreas of wild‐type and Hic‐5 KO mice. The PSCs were identified by morphological features and immunofluorescence staining for α‐SMA (Figure 5C). Cell proliferation was measured at different time‐points ranging from one to five days in cultured mice PSCs. We found that the TGF‐β‐induced cell proliferation was significantly decreased in the PSCs isolated from the Hic‐5 KO mice compared with those of the wild‐type mice (Figure 5B). After 5 days in culture without TGF‐β stimulation or 24 hours in culture with TGF‐β stimulation, the expression of α‐SMA, indicating cell activation, was significantly decreased in the Hic‐5 KO PSCs compared with the wild‐type PSCs (Figure 5C). Next, we analysed the migration ability of the cells using a wound healing and transwell migration assay. We found that the migration ability of the Hic‐5 KO PSCs was significantly decreased compared to wild‐type PSCs with or without TGF‐β stimulation (Figure 5D,E). We also found that the wild‐type PSCs exhibited high expression levels of fibrosis‐associated proteins, including α‐SMA, Col1a1, and vimentin, and that the expression levels for these proteins were significantly decreased in Hic‐5 KO PSCs (Figure 5F). Furthermore, the protein levels of NF‐κB/p65 and IL‐6 were significantly decreased in the Hic‐5 KO PSCs compared with the wild‐type PSCs (Figure 5F). These results indicated that the activation of PSCs was mediated through Hic‐5 expression and that NF‐κB/p65 may be involved in the development of CP in mice treated with cerulein.

### 3.5 Triptolide attenuates the activation of cultured PSCs by inhibiting NF‐κB/p65 expression

**Figure 6:**
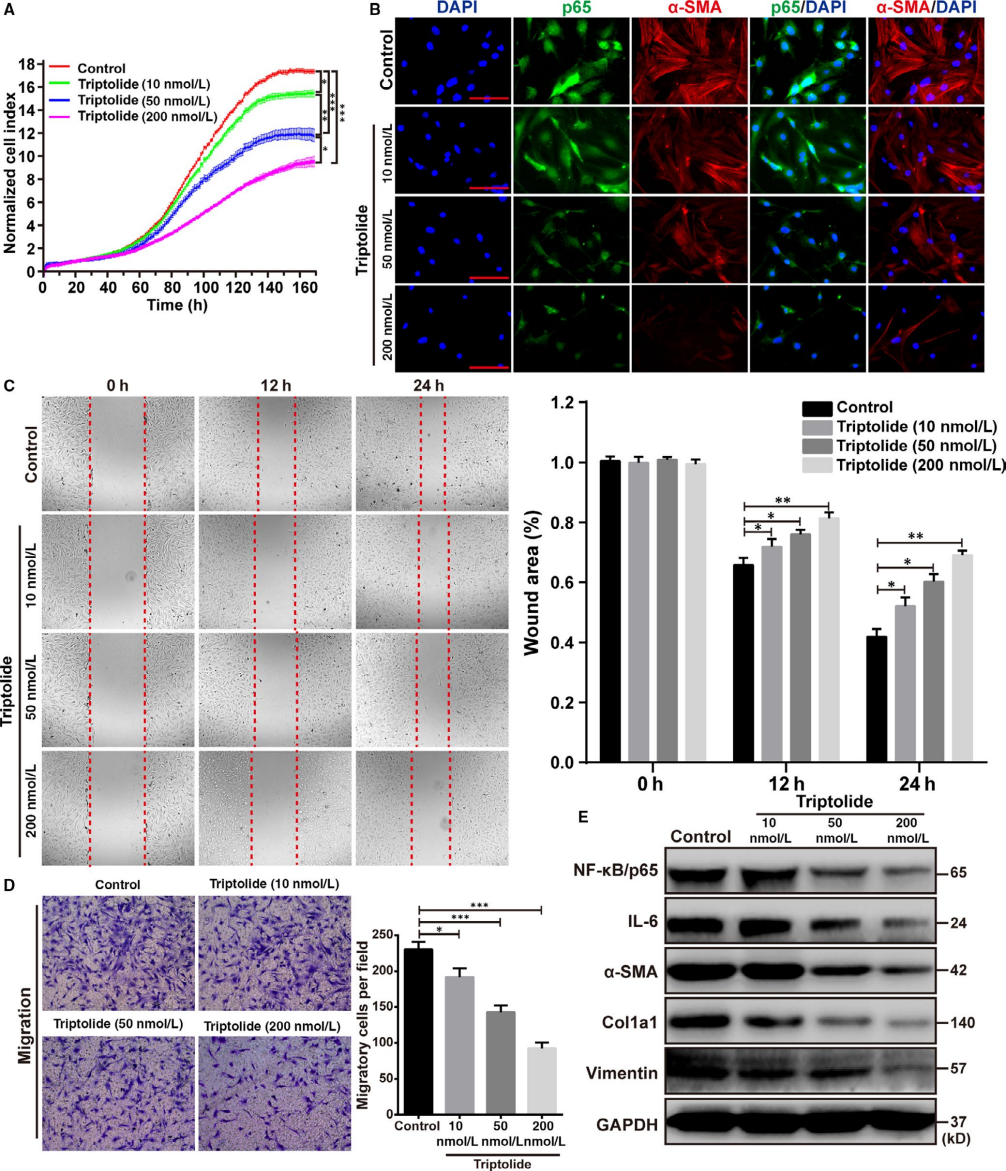
Triptolide attenuates the activation of cultured PSCs by inhibiting NF‐κB/p65 expression. A, The curve showing cell proliferation of WT PSCs that were treated with different concentrations of triptolide. B, Immunofluorescence staining for NF‐κB/p65 (green) and α‐SMA (red) in primary WT PSCs treated with different concentrations of triptolide. Scale bar, 100 μm. C, D, Representative microscopic images and quantification showing the migration of WT PSCs treated with different concentrations of triptolide. E, Representative Western blots for NF‐κB/p65, IL‐6, α‐SMA, Col1a1 and vimentin in WT PSCs treated with different concentrations of triptolide. *P < .05, **P < .01, ***P < .001, by two‐tailed, unpaired Student's *t* test. (n = 5)

To determine whether NF‐κB/p65 was involved in the development of CP, we treated primary PSCs cultures with different concentrations of the NF‐κB/p65 inhibitor triptolide, as described previously.^24^ Triptolide led to a significant and dose‐dependent reduction in the proliferation of PSCs compared to the untreated PSCs (Figure 6A). The protein levels of the related factors in PSCs were assessed by immunofluorescence. We found that the expression levels of NF‐κB/p65 and α‐SMA, which were high in control PSCs, were significantly decreased in a dose‐dependent manner in PSCs treated with triptolide (Figure 6B). In addition, analysis of the cultures for cell migration ability by the wound healing and transwell migration assay demonstrated that triptolide decreased the ability of PSCs to metastasize in a dose‐dependent manner compared to the control PSCs (Figure 6C,6). We also evaluated the protein levels of related factors in PSCs by Western blot analysis. The control PSCs exhibited high expression levels of the fibrosis‐associated proteins including α‐SMA, Col1a1, and vimentin, as well as NF‐κB/p65 and IL‐6, and the expression levels of these factors were significantly decreased in a dose‐dependent manner in the PSCs treated with triptolide (Figure 6E).

### 3.6 Triptolide alleviates cerulein‐induced CP in vivo

**Figure 7:**
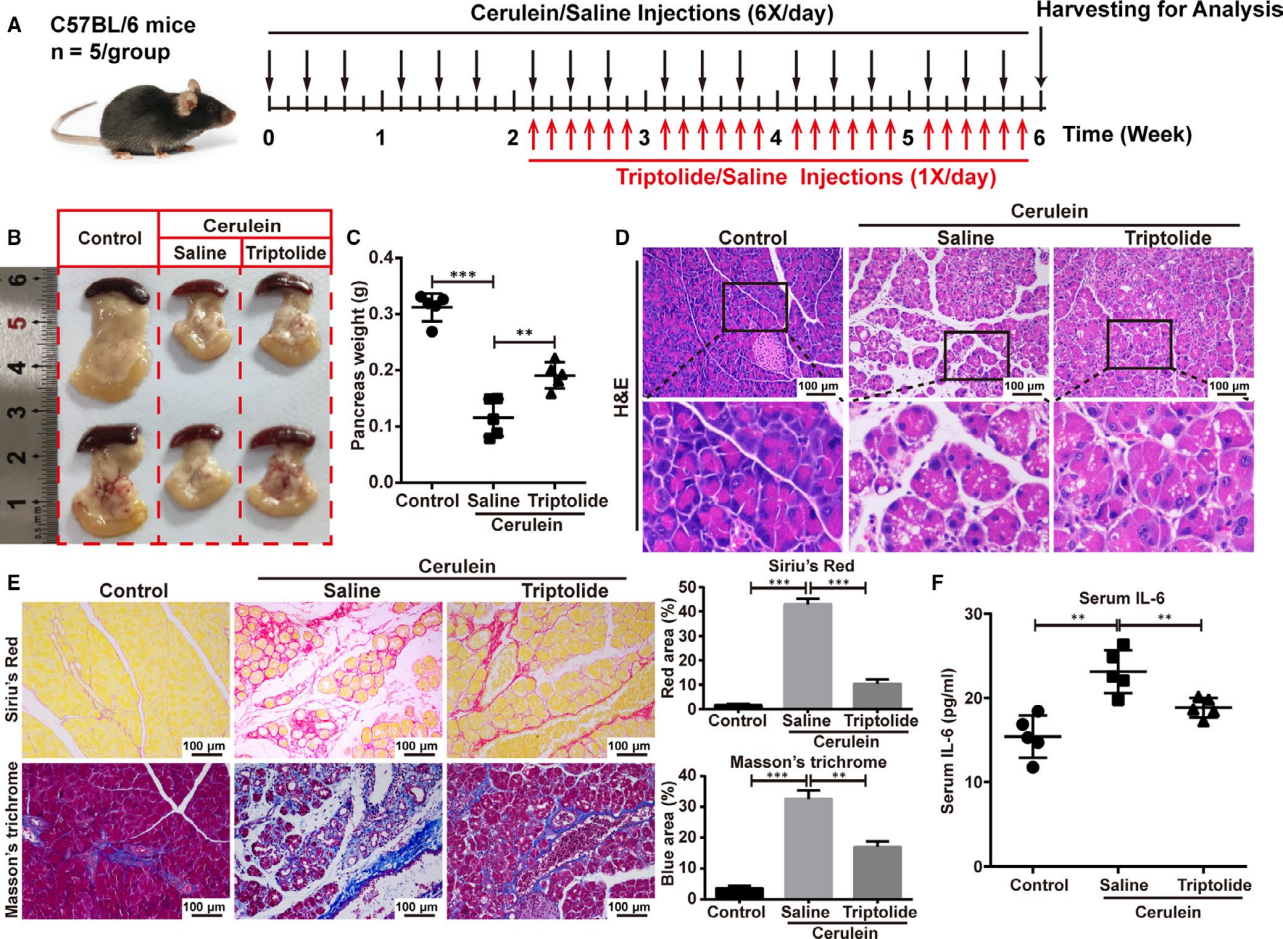
Triptolide alleviates cerulein‐induced CP in vivo. A, The schedule of mice experimental procedure is shown by a diagram. Triptolide was intraperitoneally administered at 100 μg/kg. Control group was treated with saline alone. B, Typical gross appearance of the pancreas. C, Quantification of the pancreatic weight of triptolide‐ and saline‐treated mice in which CP was induced with cerulein. D, Representative images of the H&E‐stained pancreas of the triptolide‐ and saline‐treated mice. Scale bar, 100 μm. High‐magnification images are shown below. E, Representative images and quantification of Sirius Red and Masson's trichrome staining to evaluate fibrosis. F, Serum IL‐6 levels as determined by ELISA. *P < .05, **P < .01, ***P < .001, by two‐tailed, unpaired Student's *t* test. (n = 5)

To explore the therapeutic potential of targeting NF‐κB/p65 in cerulein‐induced CP, based on the induction of CP by cerulein as described above, we started intraperitoneal injection of triptolide two weeks after the start of injection of cerulein (Figure 7A). Triptolide was administered at a dose of 100 ug/kg, once a day, six times a week, for a total of four weeks. At the end of cerulein treatment, we found that the size and weight of the pancreas of the triptolide‐treated mice with CP was significantly increased compared with the saline‐treated mice with CP (Figure 7B,7). Histological examination of the haematoxylin and eosin‐stained pancreatic sections indicated that the necrotic and inflammatory areas were significantly reduced in the triptolide‐treated mice compared with the saline‐treated mice (Figure 7D). Staining with Sirius Red and Masson's trichrome for morphometric analysis indicated that the pancreatic fibrosis after treatment with cerulein was significantly reduced in the triptolide‐treated mice compared with the saline‐treated mice (Figure 7E). We also analysed the serum level of IL‐6 by ELISA and confirmed that the level of IL‐6 was significantly lower in the peripheral blood of mice treated with triptolide than those treated with saline (Figure 7F).

### 3.7 Triptolide reduces the increased expression of cerulein‐induced pancreatic fibrosis‐related factors and NF‐κB/p65 in vivo

**Figure 8:**
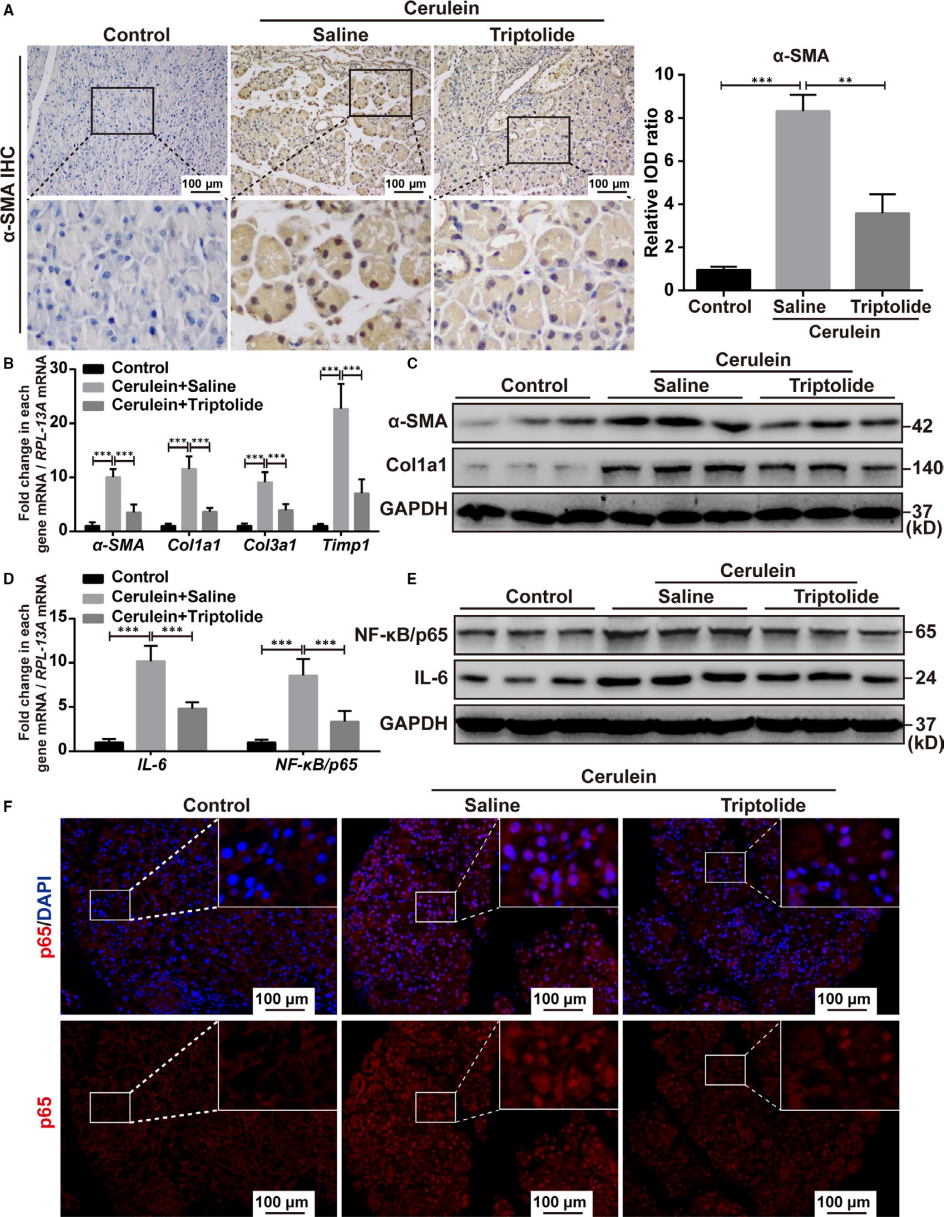
Triptolide reduces the increased expression of cerulein‐induced pancreatic fibrosis‐related factors and NF‐κB/p65 in vivo. A, Representative images and quantification of the immunohistochemistry for α‐SMA in triptolide‐ and saline‐treated mice induced by cerulein. Scale bar, 100 μm. High‐magnification images are shown below. B, D, Quantitative real‐time PCR of pancreatic tissue for α‐SMA, *Col1a1*, *Col3a1*, and *Timp1* (B), and IL‐6 and NF‐κB/p65 (D). C, E, Representative Western blots for Hic‐5, α‐SMA, and Col1a1 (C), and NF‐κB/p65 and IL‐6 (E) in the pancreas of triptolide‐ and saline‐treated mice. F, Representative images of the immunofluorescence for NF‐κB/p65 in triptolide‐ and saline‐treated mice induced by cerulein. Scale bar, 100 μm. High‐magnification images are shown below. *P < .05, **P < .01, ***P < .001, by two‐tailed, unpaired Student's *t* test. (n = 5)

Immunohistochemistry of the pancreatic sections indicated the expression of α‐SMA was significantly decreased in the pancreas of the triptolide‐treated mice compared to the saline‐treated mice (Figure 8A). We also evaluated the mRNA levels of fibrosis‐associated genes including *α‐SMA, Col1a1, Col3a1* and *Timp1* in the pancreas by qRT‐PCR and found that the expression levels of *α‐SMA, Col1a1, Col3a1* and *Timp1* were significantly lower in the pancreas of the triptolide‐treated mice than that of the saline‐treated mice (Figure 8B). In addition, Western blot analysis confirmed that the expression levels of α‐SMA and Col1a1 were significantly decreased in the pancreas of mice treated with triptolide compared with that of saline‐treated mice (Figure 8C). We evaluated the mRNA and protein levels of NF‐κB/p65 and IL‐6 in the pancreas by qRT‐PCR and Western blot analysis. The increased expression levels of NF‐κB/p65 and IL‐6 observed in the saline‐treated mice pancreas were significantly decreased in the pancreas of triptolide‐treated mice (Figure 8D,E). Furthermore, immunofluorescence and immunohistochemical analysis of the pancreas sections indicated that the expression of NF‐κB/p65 was significantly decreased in the pancreas of the triptolide‐treated mice compared to the saline‐treated mice (Figures 8F and S2A,B). We also evaluated the expression of Hic‐5 in the pancreas by Western blot analysis and found the expression of Hic‐5 was significantly up‐regulated in cerulein‐induced CP mice model, while there was no significant change after treatment with triptolide (Figure S2C). Overall, these results indicated that triptolide contributed to the improvement of cerulein‐induced CP in mice by inhibiting NF‐κB/p65.

## 4 DISCUSSION

The results of the current study demonstrate Hic‐5 as a key regulator of PSCs activation in CP. Hic‐5 was shown to be highly expressed in CP. Surprisingly, the knockout of Hic‐5 in mice attenuated CP by decreasing inflammatory cell infiltration and the expression of ECM‐related proteins and inhibiting the activation, proliferation and migration of primary mice PSCs. Simultaneously, the knockout of Hic‐5 led to a significant inhibition of the NF‐κB (p65)/IL‐6 signalling pathway and its downstream effectors including α‐SMA and Col1a1. In addition, we found that the inhibition of NF‐κB/p65 with triptolide in mice resulted in the down‐regulation of ECM‐related proteins and the alleviation of CP. As shown in Figure S3, these findings indicate that Hic‐5 is a potential therapeutic target for inhibiting PSCs activation and ECM production.

CP is characterized by massive fibroblast proliferation and deposition of ECM in the pancreas, leading to the loss of acinar cells and progressive fibrosis of the pancreas. Activation of PSCs is considered a core event in pancreatic fibrosis. By analysing the pancreatic specimens of mice with CP induced by cerulein, we are the first to show that Hic‐5 expression was significantly up‐regulated in the stroma of CP compared with the healthy pancreatic specimens. Interestingly, the expression of Hic‐5 in healthy pancreatic tissue is extremely low both in humans and mice, highlighting its potential utility as a specific therapeutic target. We found that fibrosis was greatly reduced in Hic‐5 KO mice after CP induction by cerulein, confirmed by Sirius Red and Masson's trichrome staining. As determined by the analysis of α‐SMA and Col1a1 expression levels, the knockout of Hic‐5 significantly attenuated the activation of PSCs and the production of ECM.

In response to TGF‐β, PSCs acquire a myofibroblastic phenotype, as we and others have shown previously.^30^, ^31^, ^32^ A large body of evidence from both animal and human studies demonstrate that the TGF‐β signalling pathway mediates the progression of fibrotic processes.^29^, ^33^, ^34^ Hic‐5 was originally characterized as a TGF‐β‐inducible gene. Our results indicate that quiescent PSCs exhibit extremely low expression levels of Hic‐5, activation with TGF‐β leads to a significant up‐regulation of Hic‐5 and knocking out Hic‐5 did not alter the expression levels of TGF‐β in our mice model of CP. We also found that Hic‐5 knockout resulted in the loss of proliferation and migration of PSCs with or without TGF‐β stimulation.

The role of various cells and pathways in the pancreatic microenvironment of CP, especially the role of NF‐κB signalling, has been studied extensively.^35^, ^36^, ^37^ PSCs are now recognized as the key mediators of pancreatic fibrosis. However, the role of NF‐κB in PSCs during the progression of CP has not been studied. In a study of hepatic stellate cells and liver fibrosis, the authors reported that the mechanism underlying liver fibrosis involved TNF‐α, which mediated NF‐κB activation in hepatic stellate cells and increased the ability of these cells to produce inflammatory cytokines.^38^ In the present study, we found that the levels of NF‐κB/p65 and IL‐6 were significantly increased in the cerulein‐induced CP mice model in vivo and in activated PSCs in vitro. Importantly, knockout of Hic‐5 also significantly reduced the expression levels of NF‐κB/p65 and IL‐6 in the cerulein‐induced CP model in vivo and activated PSCs in vitro.

Triptolide is a diterpenoid epoxide with the immunosuppressant activity that is an extract of the Chinese herbal medicine Tripterygium wilfordii. Triptolide was previously shown to inhibit the NF‐κB transcriptional activity and reduce NF‐κB/p65 protein levels by disrupting the interaction of NF‐κB/p65 with CREB‐binding protein.^39^ Therefore, we determined whether reducing NF‐κB/p65 levels by triptolide could alleviate CP. Interestingly, the activation, proliferation and migration of the PSCs were inhibited after treatment of PSCs with triptolide, whereas the down‐regulation of NF‐κB/p65 resulted in a significant reduction in fibrosis in mice, as demonstratedby Sirius Red and Masson's trichrome staining. The inhibition of NF‐κB/p65 not only reduced the activation of the PSCs but also led to a significant decrease in the expression of IL‐6 and downstream ECM‐related proteins (Figure 7). This finding is consistent with previous studies demonstrating that the activation of NF‐κB/p65 directly regulated IL‐6 production and promoted inflammation.^40^, ^41^ Importantly, triptolide is currently in clinical use and was shown to be beneficial in mice models of polycystic kidney disease^42^ and pancreatic cancer in vitro and in vivo. As a drug‐treated polycystic kidney disease and adjuvant therapy for human immunodeficiency virus‐1, phase III clinical trials are currently underway.^43^, ^44^ Our findings in the current study suggest that the clinically used triptolide doses might also provide benefit in patients with CP.

In conclusion, the current study using both in vitro and in vivo approaches is the first to demonstrate the role of Hic‐5 in PSCs expression and promotion of CP. Hic‐5 is critical for maintaining the phenotypic characteristics and activation of PSCs. These results provide evidence for Hic‐5 as a key target for maintaining the phenotype and activation of PSCs. Our studies in Hic‐5 KO mice demonstrated the regulatory role of Hic‐5 on PSCs activation and that targeting Hic‐5 could alleviate pancreatic fibrosis and CP. In addition, we found that the lack of Hic‐5 reduced the expression of NF‐κB/p65, indicating that Hic‐5 deletion in PSCs inhibited the NF‐κB (p65)/IL‐6 signalling pathway and alleviated CP. Overall, these results suggest that the development of novel therapeutic strategies such as NF‐κB p65 inhibitors that aim at decreasing Hic‐5 and/or NF‐κB/p65 can improve precision medicine approaches for the treatment of CP.

## CONFLICT OF INTEREST

The authors declare no conflicts of interest.

## AUTHOR CONTRIBUTIONS

Wenguang Fu and Tiancheng Liang designed the research; Hao Chen and Peng Tan performed the experiments; Baolin Qian and Zhiwei Huang analysed data; Hao Shi and Yichao Du wrote the manuscript; and Ankang Wang and Shiyao Huang refined the manuscript.

## Data Availability

The data that support the findings of this study are available from the corresponding author upon reasonable request.
