# Supplementary material for: Hic‐5 deficiency protects cerulein‐induced chronic pancreatitis via down‐regulation of the NF‐κB (p65)/IL‐6 signalling pathway
Source: J Cell Mol Med. 2019 Dec 3;24(2):1488–503. doi: 10.1111/jcmm.14833 (PMC6991662; doi:10.1111/jcmm.14833)
Supplement: Supplementary file 1 [file JCMM-24-1488-s001.docx]

**Supplementary Information**


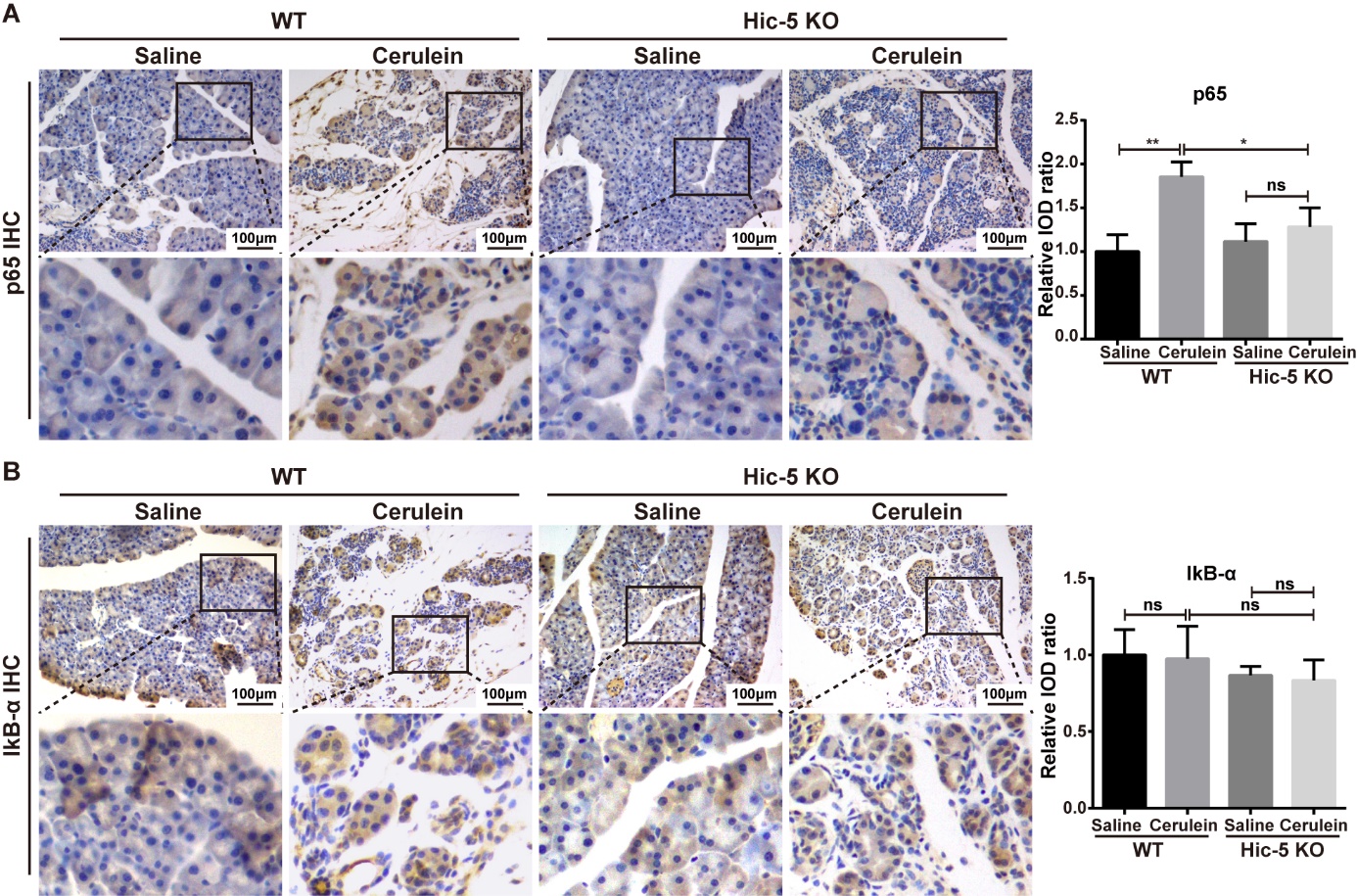


**Figure S1.** Expression of NF-κB/p65 and IκB-α in pancreatic tissues. A, B) Representative images and quantification of the immunohistochemistry for NF-κB/p65 (A) and IκB-α (B) in WT mice and Hic-5 KO mice with CP induced by cerulein. Scale bar, 100 μm. High-magnification images are shown below.


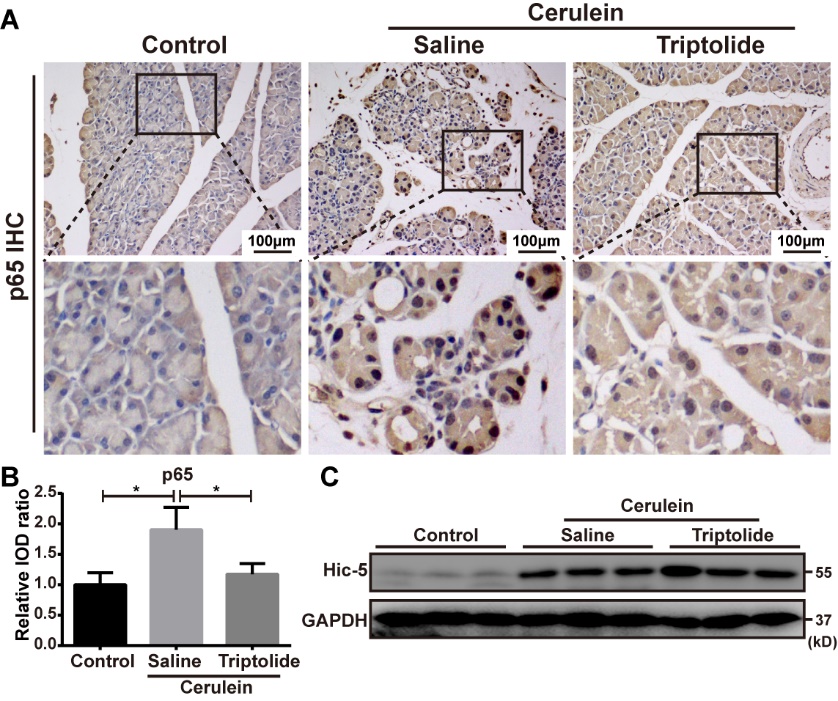


**Figure S2.** Expression of NF-κB/p65 and Hic-5 in pancreatic tissues. A, B) Representative images (A) and quantification (B) of the immunohistochemistry for NF-κB/p65 in triptolide- and saline-treated mice induced by cerulein. Scale bar, 100 μm. High-magnification images are shown below. C) Representative Western blots for Hic-5 expression in s triptolide- and saline-treated mice induced by cerulein.


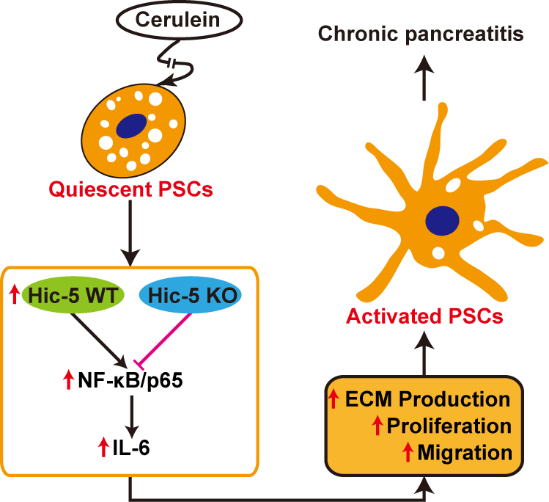


**Figure S3.** A signal transduction diagram showing that Hic-5 involved in cerulein-induced chronic pancreatitis.
